# Spin-Current and Spin-Splitting in Helicoidal Molecules Due to Spin-Orbit Coupling

**DOI:** 10.1038/srep23452

**Published:** 2016-03-24

**Authors:** R. A. Caetano

**Affiliations:** 1 Departamento de Física, Universidade Federal do Paraná, Caixa Postal 19044, 81531-990, Curitiba, PR, Brazil

## Abstract

The use of organic materials in spintronic devices has been seriously considered after recent experimental works have shown unexpected spin-dependent electrical properties. The basis for the confection of any spintronic device is ability of selecting the appropriated spin polarization. In this direction, DNA has been pointed out as a potential candidate for spin selection due to the spin-orbit coupling originating from the electric field generated by accumulated electrical charges along the helix. Here, we demonstrate that spin-orbit coupling is the minimum ingredient necessary to promote a spatial spin separation and the generation of spin-current. We show that the *up* and *down* spin components have different velocities that give rise to a spin-current. By using a simple situation where spin-orbit coupling is present, we provide qualitative justifications to our results that clearly point to helicoidal molecules as serious candidates to integrate spintronic devices.

One of the main bottlenecks for the realization of spintronic devices is the difficulty in appropriately selecting electronic spin-polarization, without the need of an external magnetic field. In this direction, most of the progress to the date is based on inorganic heterostructured materials with large Rashba spin-orbit coupling, allowing to control the spin degree of freedom with voltage gates on top of the heterostructures^1^,^2^,^3^,^4^. However, the use of organic materials is a tantalizing route towards spintronics since they can offer certain advantages in relation with inorganic materials^5^,^6^. For instance, organic materials are inexpensive, mechanically flexible, chemically interactive and easy to fabricate^7^. On the other hand, the light elements of organics lead to low spin-orbit coupling and, thus, spin-dependent properties are very unlikely. Despite the low spin orbit coupling, an unexpected spin-selectivity of electrons injected from ferromagnetic electrodes has been demonstrated^8^,^9^. Recently, the observation of spin-dependent electrical properties in organic chiral molecules in the absence of any magnetic material has been reported in the literature, triggering a strong interest in these materials^10^,^11^,^12^,^13^,^14^. There are two main theoretical approaches in order to explain this unconventional spin-selectivity. The first one, based on scattering theory, includes spin-orbit coupling originated from a helical potential, addressing properly experiments where the energies of the emitted electrons are well above the energies of the molecular orbitals in DNA^15^,^16^. Therefore, it can be understood as scattering processes by an external helicoidal potential. The experiments reported in Reference^11^ are well described by this point of view. The second approach is suitable to describe experiments reported in Reference^13^, where the electrical properties of a self assembled DNA monolayer is probed by using a two terminal setup. Gutierrez *et al.* have formulated a quantum transport model which includes the spin-orbit coupling due to the presence of a helicoidal external electric field originated from charge accumulation along the helix^17^,^18^ and a strongly spin-dependent current has been found. In these formulations, however, spin-orbit coupling alone is not enough to promote spin-dependent electrical properties. Artificial violation of time reversal symmetry^17^, two energy levels per site and asymmetries in the electronic-coupling elements between the different channels^18^ are mandatory ingredients in order to find spin-dependent electrical properties. In the same direction, Guo and Sun have formulated a model which consider an electron moving in a double helical path under action of a helicoidal electrostatic potential^19^. It has been shown that to obtain such high spin selectivity, however, is necessary to include a mechanism to break the electronic phase through the interaction with virtual Büttiker leads. The authors also show that double-strand structures are necessary to get spin polarization. In the absence of any of these conditions, no spin-selectivity is obtained. Very recently, it has been theoretically demonstrated that the presence of spin-orbit coupling in helicoidal molecules, further than to induce spin selectivity, strongly enhances the electrical transmission. The authors also show that, in order to observe such spin polarization, a strong electrical dipole filed must be present^20^. Still with the intention of justifying the experimental results, Matityahu *et al.* have considered a simple model where spin polarization is obtained by combining spin-orbit coupling and breaking of the unitarity of the scattering matrix^21^.

Concerning the electronic properties, Medina *et al.* have formulated a continuum model, which can be exactly solved, for chiral molecules including spin-orbit coupling. The eigenvalues/eigenvectors spectrum reveals the appearing of two Kramer doublets transport channels separated by a gap which proportional to the spin-orbit coupling constant. The authors also show the velocities of opposite spin orientation are different, leading to spin and charge current when split Kramers doublets are considered^39^. Nevertheless, although the work by Medina sheds light on the nature of the eigenstates revealing the important features as the ones described above, a more realistic behavior of the electron is obtained by analyzing the dynamics of a wave packet. It allows us to simulate different experimental conditions and, thus, to explore the physical features of each scenario. In what follows, we shall describe the electron by a wave packet and it will be possible to establish what are the conditions to the appearing of a persistent spin-current and the very desirable spin-splitting. It is worth to say that we are interested in the situation where there is spin-transference without charge transport. Thus, we follow the time evolution of an initial unpolarized electronic wave packet moving in a helicoidal molecule, DNA as a representative example, and we show that, although the spin-orbit coupling derived by Guo and Sun^19^ does not lead to an unbalanced spin polarized current, it is enough to promote a spatial separation between the |↑〉 and |↓〉 spin components. We show that, due to the spin-orbit coupling, the opposite spin polarization evolve with opposite velocities along the molecule, leading to a finite spin-current. This spatial separation of the spin components suggests that it is possible to use of helicoidal molecules as spin filters in spintronic devices. While previous studies demonstrated that spin-dependent properties were found in double stranded molecules (or in systems with two energy levels per site), we show that the spatial spin separation can also be observed in single stranded molecules with only one level per site, enabling a wider range of potential molecules for spintronic applications. It is important to stress that all models mentioned above require a helicoidal symmetry, which plays a crucial role in the process. In fact, the influence of the chiral symmetry was previously considered in many different contexts, such as the study of the electronic states in curved wires^22^, chiral nanotubes^23^,^24^ and general curvelinear coordinates Schröringer equation^25^. However, the helicoidal geometry itself does not lead to spin-orbit coupling. The central ingredient in order to have spin-orbit coupling is an electrostatic potential which, in the case of DNA molecules, is originated from accumulated charges along the helix^26^.

## Model and Results

**Figure 1:**
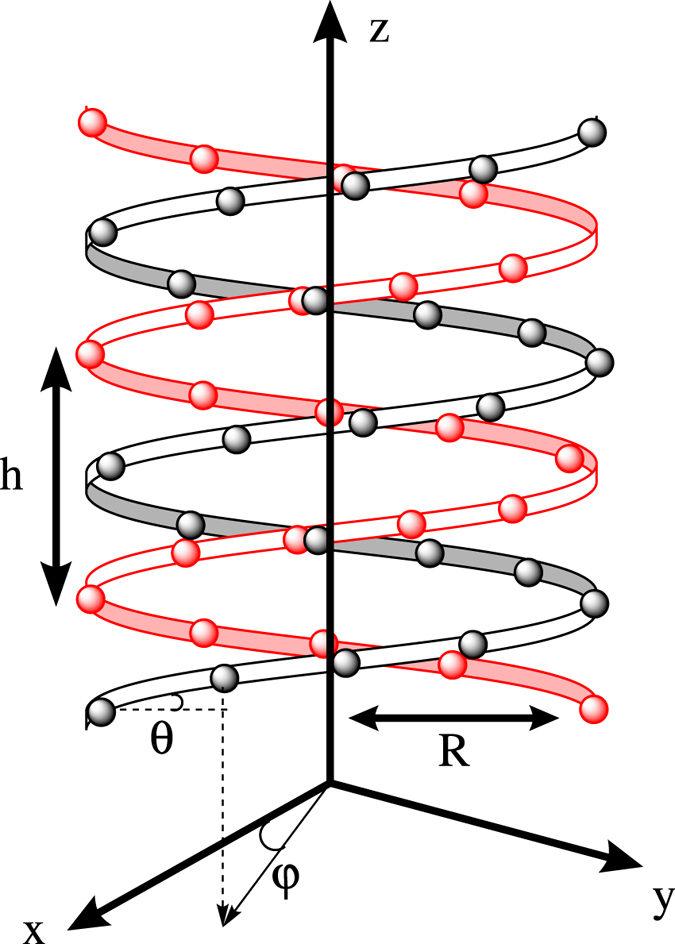
Illustration of a double-strand DNA molecule with radius R, helix angle *θ* and pitch h. The nucleobases are represented by the circles. The arc length between two neighboring nucleobases is *l*_*a*_, which satisfies the relation *l*_*a*_ cos*θ* = *R*Δ*φ* and *l*_*a*_ sin*θ* = Δ*h*, where Δ*φ* is the twist angle and Δ*h* is stacking distance between two nucleobases. In order to mimic the double-strand DNA molecule, we set Δ*h* = 0.34 nm, Δ*φ* = *π*/5, *h* = 3.4 nm, *R* = 0.7 nm, *θ* ≈ 0.66 rad and *l*_*a*_ ≈ 0.56 nm.

Let us start considering one electric charge, which can be controllably introduced by an AMF tip^27^,^28^,^29^ or through optical exitation^46^ under influence of an electrostatic potential, *V*, moving along a double stranded helicoidal molecule, illustrated in Fig. 1. It has been shown that the spin-orbit coupling felt by the electron is described by the Hamiltonian^19^,^30^:


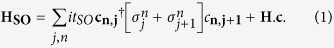


where 
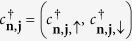
 is the creation operator of a spinor at site *j* of the *n*^*th*^ strand of a double strand molecule, 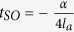 is the spin-orbit-coupling constant, 
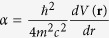
 and 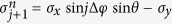

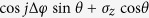
. *l*_*a*_, Δ*φ* and *θ* are the arc length, the twist angle between first-neighbor sites and the helix angle, respectively. They are related by: *l*_*a*_ cos *θ* = *R*Δ*φ* and *l*_*a*_ sin *θ* = Δ*h*, where *R* is the radius of the helix and Δ*h* is the stacking distance between neighbor sites. **H.c.** is the hermitian conjugate. For DNA molecules, where the two strands have a *π* phase difference, we have that 
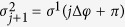
. The total Hamiltonian is given by:


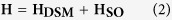


with 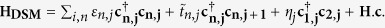 being the usual double-strand Hamiltonian, where *η*_*j*_ is the inter-strand hopping integral at site *j*,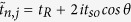 and *t*_*R*_ is the intra-strand hopping overlap integral. The solution of Equation (1) is given by a four component spinor of the form: *ψ* = (*ψ*^(1)↑^, *ψ*^(1)↓^, *ψ*^(2)↑^, *ψ*^(2)↓^)^*T*^, where *ψ*^(*n*)*σ*^ is the wave function component on the *n*^*th*^ strand with spin polarization *σ*. The time evolution is governed by the Schrödinger equation, which is given, in terms of the Wannier amplitudes, by:


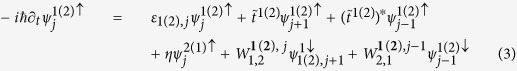



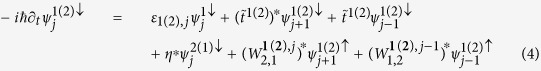


here, 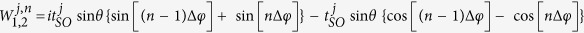. One must notice that 
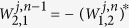
, thus, the time reversal symmetry is preserved.

**Figure 2:**
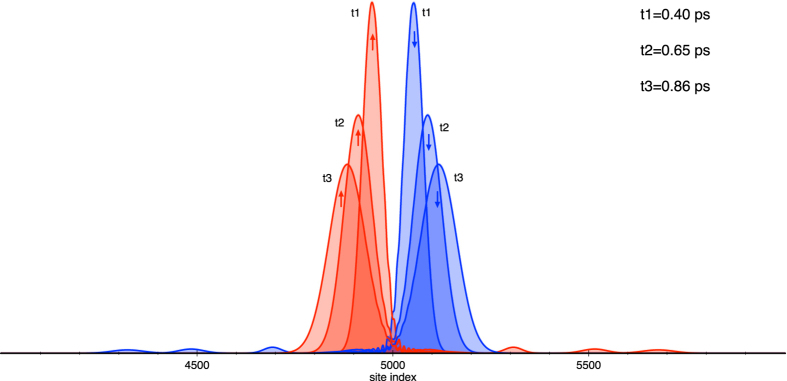
Time evolution of an initially gaussian wave packet, with l = 30, for different times: *t*_1_ = 0.4 ps, *t*_2_ = 0.65 ps and *t*_3_ = 0.86 ps. The wave packet evolves in a double strand molecule described by the parameters: 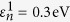, 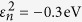, 
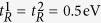
, *η* = 0.3 eV, *θ* = 0.66 and 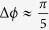 and *t*_*SO*_ = 0.03 eV. The *up* and *down* spinor components are represented by red and blue, respectively.

We have used eighth-order Taylor expansion in order to solve Equations (3) and (4) simultaneously with time step Δ*t* ~ 10^−3^ fs. This time step is small enough to keep the spinor normalized during the whole simulation. We follow the time evolution of an initially spin-unpolarized gaussian wave packet with width equal to *l* = 30 nucleobases, placed at the center of the molecule: 
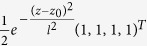
. For double strand DNA molecules, we set the parameters: 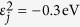, 
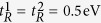
, 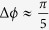, *η* = 0.3 eV, *θ* = 0.66 and 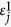
31. It is worth stressing that the effects reported here are strongly robust to chosen of the parameters (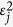, 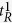, 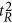, 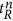, *η*). Figure 2 shows the wave packet at three different time steps: *t*_1_ = 0.4 ps, *t*_2_ = 0.65 ps and *t*_3_ = 0.86 ps where we have used the spin-orbit constant equal to 0.03 eV, which is one order of magnitude smaller than the hopping parameter, 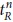
19. Despite the fact that the spin-orbit coupling does not produce any polarization, it can be observed that the spin *up* and *down* components move in opposite directions, leading to a spatial separation between spin polarizations.

### Drifting Velocity and Spin Current

**Figure 3:**
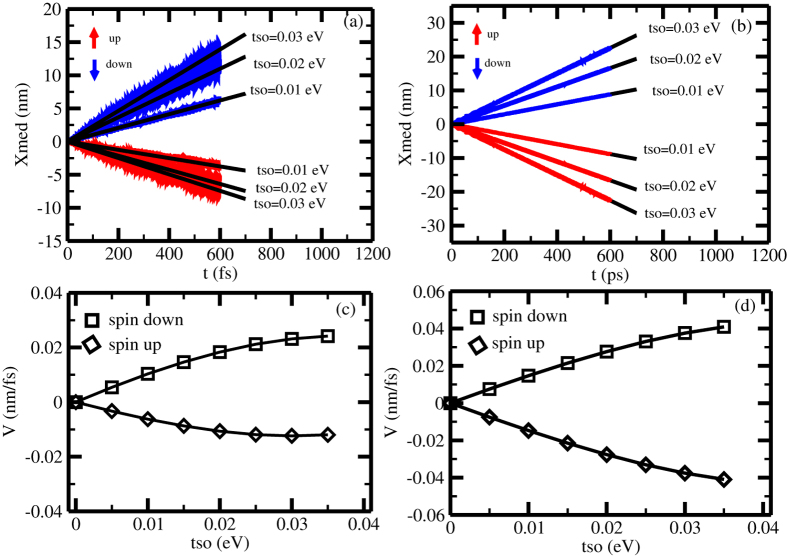
Time evolution of the mean position, defined by: 
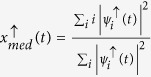
 and 
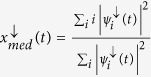
, of an initially gaussian wave packet, in a double strand molecule, with width (**a**) *l* = 1 and (**b**) *l* = 30 for three spin-orbit coupling constant: *t*_*so*_ = 0.01, *t*_*so*_ = 0.02 and *t*_*so*_ = 0.03 eV. (**c**,**d**) are, respectively, the mean velocity of the wave packet components for the cases studied in (**a**,**b**).

In order to understand this phenomenon, we shall show that the presence of spin-orbit coupling adds an extra term to the canonical momentum. This extra term depends on the spin polarization and the canonical momentum becomes different from the kinetic momentum. Quantitative information about the separation between the spin components can be obtained by looking at the dynamics of the wave packet. One convenient quantity to be studied is the mean position of each component of the spinor, defined by: 
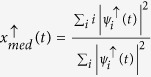
 and 
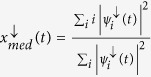
. Figure 3(a,b) show the time evolution of the mean position of the spin *up* and spin *down* of an unpolarized initial wave packet, with *l* = 1 and *l* = 30, respectively.

By observing Fig. 3(a,b), one can see clearly that each spin component has a net movement in a well defined direction. Furthermore, the mean velocity of the narrower initial wave packet, shown in Fig. 3(c), is smaller than the mean velocity of the wider initial wave packet, shown in Fig. 3(d). It is interesting to notice that the absolute value of the velocity for the *up* component is different than the velocity of *down* component when a narrow initial wave packet is considered. On the other hand, the velocity of both components has the same magnitude for the wider initial wave packet (l = 30). A qualitative justification to this behavior will be provide by exploring the simplest situation where spin-orbit coupling is present.

From a purely quantum mechanical point of view, this phenomenon can be seem as a flux of angular momentum, without a simultaneous flux of electronic charge. Traditionally, the quantity used to quantify this angular momentum flux is the spin-current^32^,^33^,^34^, defined as 
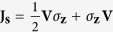
35, where **V** is the velocity operador given by: 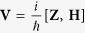 with **Z** being the position operator. Thus, the mean spin-current, *I*_*s*_ = Tr (*ρ***J**_*s*_), where *ρ* is the density matrix, is given by:


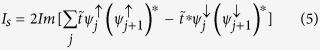


In fact, the spin-current is one of the most important physical quantities in spintronics and has been extensively studied from fundamental and technological perspective, such as the spin Hall effect^36^,^37^ and spin precession^2^,^38^.

**Figure 4:**
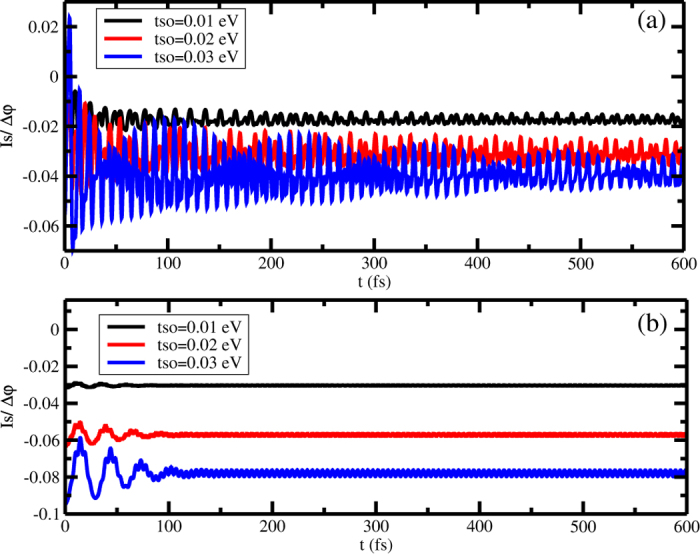
(**a**,**b**) show the time evolution of the spin current for an initially unpolarized gaussian wave packet in a single strand helicoidal molecule, with *l* = 1 and *l* = 30, respectively, for spin-orbit coupling, *t*_*so*_, equal to 0.01, 0.02 and 0.03 eV.

Figure 4(a) shows the spin-current for an initial gaussian wave packet with width *l* = 1 for three spin-orbit coupling strength: *t*_*so*_ = 0.01, *t*_*so*_ = 0.02 and *t*_*so*_ = 0.03 eV and Fig. 4(b) shows the spin-current for a initially gaussian wave packet with *l* = 30 for the same spin-orbit coupling constant of Fig. 4(a). It can be observed that, in both cases, there is a time persistent spin-current for any spin-orbit coupling strength. As one could anticipate by looking at Figs 3 and 4 shows that the magnitude of the spin current is smaller for the narrower initial wave packet than for the wider initial wave packet. A qualitative understanding of Fig. 4 can be obtained by analyzing the simplest case of spin orbit coupling in two limits, namely, a totally localized initial wave packet and a totally spread initial wave packet.

It is worth to emphasizing the differences between the results presented in the present manuscript and those ones reported in References^17^,^18^,^19^. The results in refs 17, 18, 19 are all based on charge transport followed by a spin polarization. This means, they show how electrons with opposite spin polarization flow differently through the helicoidal molecules when a voltage is applied. The observation of such polarized current requires, however, further ingredients besides spin-orbit coupling. For example, in reference^17^, it is necessary to break time reversal symmetry to observe the spin polarization. Loss of the electronic phase and spin memory, due to inelastic scattering, is also a mandatory ingredient in ref. 19. It was shown in ref. 18 that polarized current in double strand molecules can be obtained without electronic phase breaking or spin memory, however, the electronic coupling parameters and the spin-orbit coupling must be asymmetric in order to observe sizable spin polarized current. On the other hand, spin-orbit coupling leads to spin transfer (which is not followed by a charge transfer) without the need to include any other ingredients.

### Analytical consideration for limiting cases

It is convenient to analyze the case of a single stranded molecule with one energy level per site. Doing this: (1) all physically important features can be more easily understood in single strand molecules and (2) contrary to the polarization effects observed in the References^17^,^18^,^19^, the splitting of the spin *up* and *down* components can be observed even for single strand with one level per site. In this section, we set *ħ* = 1. Under these conditions, the initial wave packet is given by:


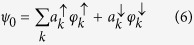


where 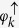 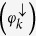 is an eigenstate of the **H** [Equation (2)] associated with the eigenvalue 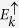 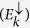. The expansion coefficients 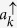 and 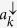 are given by: 
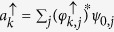
 and 
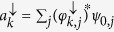
, where 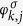 and 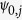 are the value of the eigenvector 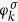 and the initial wave packet at the *j*^*th*^ site. Due to the periodic nature of the potential, both eigenvectors can be written as Bloch states:


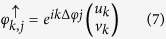


and


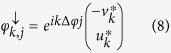


where *u*_*k*_, *v*_*k*_ are functions with periodicity equal to the potential.

Combining these properties, the time evolution of the spin components can be written as:


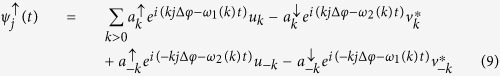


and


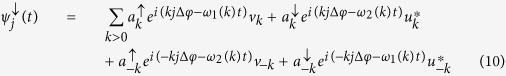


where we have explored the time reversal symmetry by doing 
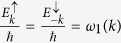
 and 
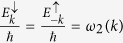
. At this point, the knowledge of the eigenstates and eigenvalues of Equation (2) is necessary. Using the Ansatz^39^,^40^:


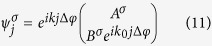


where *k*_0_ is determined by the periodicity of the spin-orbit coupling potential. By applying Equation (11) for **H** in Eq. (2), it is possible to find the eigenvalues of **H**. However, the expression for the eigenvalues is rather complicated for a general *k*_0_ and no useful analytical expressions can be obtained. On the other hand, the simple case where *k*_0_ = 0 captures many important features. In this case, the eigenenergies are:


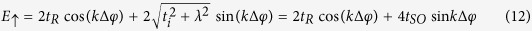



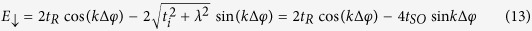


here, we have defined *t*_*i*_ = 2*t*_*so*_ cos*θ* and *λ* = 2*t*_*so*_ sin*θ*. The eigenvector associated with *E*_↑_ and *E*_↓_ are, respectively:


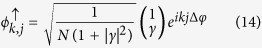



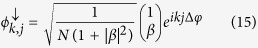


with 
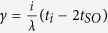
 and 
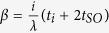
. We shall apply these results for two interesting limiting cases: (*i*) a totally localized initial wave packet and (*ii*) a completely delocalized initial wave packet.

### Localized initial wave packet

The first case that we analyze is the case where the two components of the initial wave packet are localized on a single site, *j*_0_, on the molecule: 
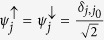
. Without loss of generality, let us consider that the initial wave packet is localized at *j*_0_ = 0. In this limit, the coefficients *a*_*k*_’s in equations 9 and 10 are:


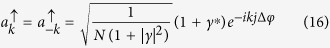



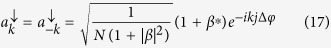


One should notice that Equations (16) and (17) do not depend on *k*. This means that the Equations (9) and (10) have contributions associated with all *k*’s within the first Brillouin zone and can be written as:


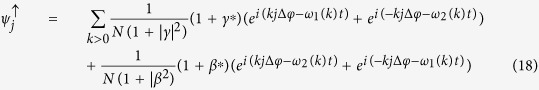



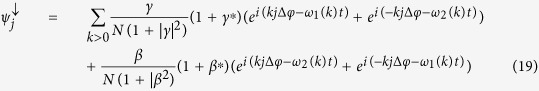


The spin current, thus, is given by:


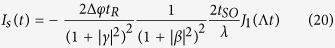


where *J*_1_(*x*) is a first-order Bessel function of the first kind and Λ = 8*t*_*SO*_. By examining Equation (20), one can see that the spin-current oscillates around zero and, thus, no net spin-current is observed. Physically, this fact can be understood as follow: The narrow initial wave packet is written as a sum of many terms associated with the wave vector *k* (in this limit case, the sum contains an infinity numbers of terms). Thus, the spin components of the initial wave packet have terms that can be identified as plane wave traveling in opposite directions (see Equations (18) and (19)) that lead to a vanishing spin-current.

### Delocalized initial wave packet

The other limit we must study is the one where the initial wave packet is totally delocalized over the whole system: 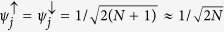, for all *j* in the interval 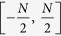, where *N* + 1 is the number of sites. Since 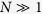, we have made: 2(*N* + 1) ≈ 2*N*. The coefficients in Equations (9) and (10) are:


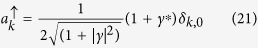



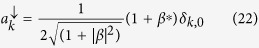


Therefore, the spinor components can be written as:


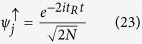



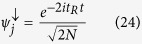


One can show that 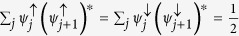 and, thus, the spin-current is:


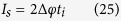


It is interesting to notice that only the state associated with *k* = 0 is present. However, contrary to what one could have expected, the spin current is nonzero, meaning that there is a relative velocity between |↑〉 and |↓〉 states even for *k* = 0. When spin-orbit coupling is taken into account, the canonical momentum is not equal to the kinetic momentum, which is similar to the case of an electron moving in a magnetic field. One can see this clearly by looking at the phase velocity of the electron in both energy bands:


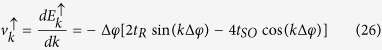


and


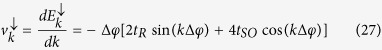


One can see from Equations (26) and (27) that, even for *k* = 0, the velocity is nonzero. Furthermore, the additional velocity introduced by the spin-orbit coupling is opposite for different spin polarization.

At this point, we can qualitatively justify the behavior shown in Fig. 3. In particular, Fig. 3(c) shows that the packets with opposite polarization move in opposite directions; however, the magnitude of their velocities is different. Consider an extremely narrow initial wave packet with nonzero width. One can construct such wave packet by summing over every wave vector *k* (as done in Equations (18) and (19), except over a specific wave vector *k*_0_:


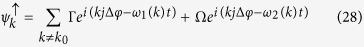



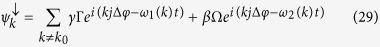


where 
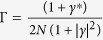
 and 
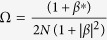
. The mean velocity of the initial wave packet given by equations 28 and 29 can be obtained by: *Tr*(*ρ***V**):


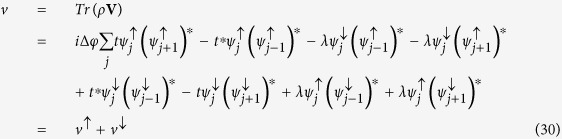


One can identify the four first terms in equation 30 as the velocity of *up* component of the spinor, *v*^↑^, and last four terms can be identified as the velocity of the *down* component, *v*^↓^. Clearly, either *v*^↑^ or *v*^↓^ can be complex number. The physical meaning of the imaginary part of *v*^↑^ and *v*^↓^ is the population changing in each polarization and the real part gives variation of the position of the center of mass of the packet. In this sense, we are interested only in real parts of *v*^↑^ and *v*^↓^, which are given by:


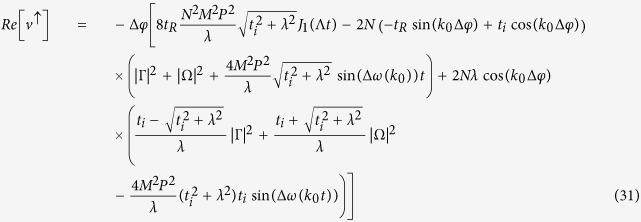



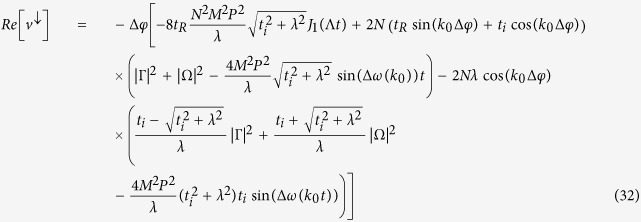


where 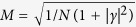 and 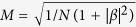. Indeed, the magnitude of the two velocities is different. From another perspective, it is possible to understand this fact by considering that, in order to get an unpolarized initial wave packet a little wider than a delta function, it is necessary to remove at least one state from each band. As shown above, the phase velocities are different in each band. Thus, the velocity of the *up* component must be different from the velocity of *down* component. This behavior can be clearly seen in Fig. 3(c). On the other hand, the velocities of both polarization are the same for an initial wide wave packet [Fig. 3(d)]. As was already pointed out in Equations (23) and (24), only the wave vector *k* = 0 is present. Thus, the velocities of the spinor components are:


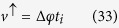



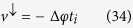


Thus, the velocities of spinor components are time independent and are opposite but equal in magnitude.

### Spin Spatial Separation

Another very desirable property of materials that are candidates to integrate spintronics devices is the ability to select a given spin polarization without the need of an external magnetic field. These components are known as spin-filters and play a central role on logical operations in spintronics. In this sense, a spatial separation between two polarizations fulfills this need. In order to quantify the degree of separation of the spin components in the wave packet, we look at the the projection of the *up* component on the *down* component of the wave packet: 
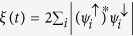
. In the limit where the two components are in the same region, *ξ*(*t*) is one. On the other hand, if the two components are completely separated, *ξ*(*t*) is zero.

**Figure 5:**
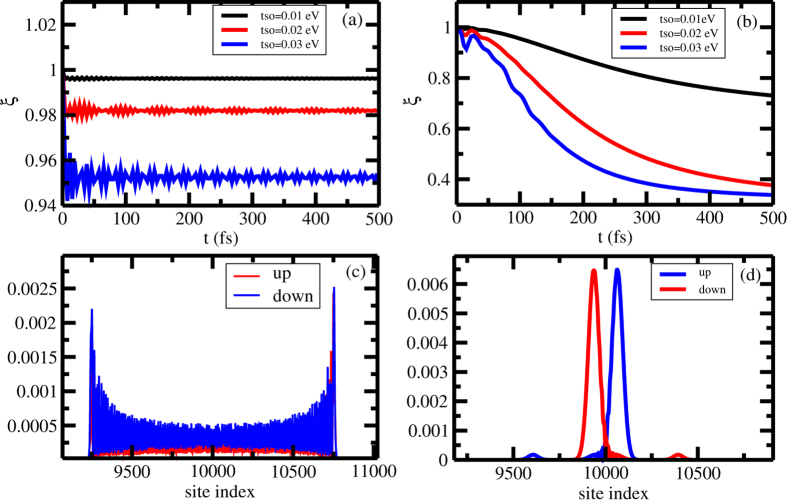
(**a**,**b**) show the time evolution of *ζ* function for an initially unpolarized gaussian wave packet in a single strand helicoidal molecule, with *l* = 1 and *l* = 30, respectively, for spin-orbit coupling, *t*_*so*_, equal to 0.01, 0.02 and 0.03 eV. (**c**,**d**) show, respectively a snapshot of the absolute squared of the wave packet at *t* = 0.49 ns of the initial wave packet with *l* = 1 and *l* = 30 and *t*_*so*_ = 0.03 eV.

Once more, we turn our attention to a much simpler system: the single strand molecule, with a single orbital per base. Figure 5(a) shows the time evolution of the *ζ* function of an initially gaussian wave packet with width equal to 1 for three values of spin-orbit coupling: *t*_*so*_ = 0.01, 0.02 and 0.03 eV. One can observe that, even for the largest spin-orbit coupling constant, there is not a substantial separation between *up* and *down* components. The dominant effect here is the spreading of both components. This spread can be directly seen by looking at the electronic wave packet in a system with spin-orbit constant equal to 0.03 eV at *t* = 0.49 ps, as shown in Fig. 5(c). It can be noticed that both components occupy nearly the same region. Qualitatively, this behavior can be understood by looking at Equations (31) and (32). Albeit the velocities of the spinor components are different, the dominant terms are the one which involve the first order Bessel function. Thus, the mean position of each component performs an oscillatory movement around the initial position and no separation can be observed. On the other hand, for a wider initial wave packet, there is a clear spatial separation with the increase of the spin-orbit coupling, as Fig. 5(b) shows. Again, this separation between the components can be seen by looking at the wave packet. Figure 5(d) shows the components of the wave packet at *t* = 0.49 ps in a system with spin-orbit coupling equal to 0.03 eV. Clearly spin *up* and spin *down* wave packet have different spatial functions. Likewise done previously, this action can be understood in the lights of Equation (33) and (34). Since both components have opposite constant velocities, the mean position of each components moves away from each other, leading to a decreasing of the *ξ* function.

## Conclusions

In conclusion, we have considered single and double strand helicoidal molecules in a presence of Rashba spin-orbit coupling. We have demonstrated that, within the model proposed by Guo and Sun^19^, due to the spin-orbit coupling, a spatial separation between spin *up* and *down* of an initially unpolarized wave packet emerges. We also have shown that the spin-orbit coupling gives rise to a spin-current. An important feature induced by the spin-orbit coupling it the fact that it makes the canonical momentum different from the kinetic momentum. Furthermore, each component of the spinor has an extra and opposite term added to the velocity. With the use of the wave packet to describe the electron, it was possible to study different scenarios and to establish what are the experimental conditions to get an efficient spin separation and high spin current. In order to get an efficient spatial separation and higher spin-current, one should prepare, as the initial state, a well defined wave packet in the momentum space. It is worth to call the attention that the results shown here do not depend on a break of phase, however, its quantitative importance needs a further investigation. Furthermore, different from electrical properties, which are based on an unbalance of between *up* and *down* polarization, the properties shown here are also present in single strand molecules with only one level per site. Thus, single strand molecules with one site per site (single strand DNA, for instance) can also be considered for technological applications. It is important to keep in mind that, formally there is no need of a net polarization in any system presented here. There is only a spacial separation between spin *up* and *down*. Thus, our results do not violate the statement done by Guo and Sun that forbids spin polarization in single strand molecules^19^. From the experimental point of view, the detection of spin current is one of the most important and defiant issue in spintronics. Most of the progress in this field uses spin Hall effect to detect spin current. However, despite the spin accumulation at the edges of the sample, it does not produce a measurable electrical signal^41^,^42^. However, it has been proposed that spin current can be electrically probed by using the Inverse spin Hall effect, which converts spin current into charge current, in ferromagnetic materials^43^. Very recently, a new experimental technique has been reported in the literature allowing to measure spin-current in organic semiconductor^44^. We believe that something in this direction could be used in order to measure pure spin current in helicoidal molecules. We hope that, due to the great potential of organic chiral molecules, our work can stimulate experimental progresses in this direction.

## Method

Let us derive the expression of the spin current for an initial completely localized wave packet [equation (20)] Each term of Equation (5) can be calculated by using the expressions (18) and (19). We start calculating the first term of the sum in Equation (5):


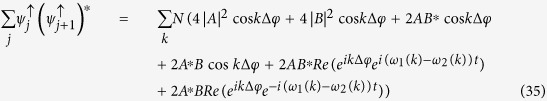


where *A* = 2/*N*(1 + |*γ*|^2^(1 + γ^*^)) and *B* = 2/*N*(1 + |*β*|^2^(1 + β^*^)). In the thermodynamic limit, the sum in *k* can be replaced by an integral: 
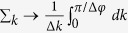
, thus, Equation (35) can be written as:


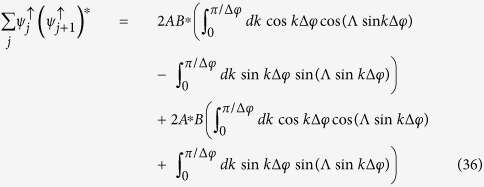


By using the relations 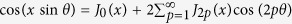 and 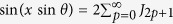

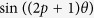
45, where *J*_*n*_(*x*) is the first kind Bessel function of order *n*. In this way, the first term of Equation (5) is:


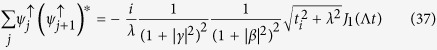


Similarly, one can show that the second term of Equation (5) is given by: 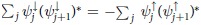 and for the most localized initial wave packet, the spin current [equation (5)] is given by:


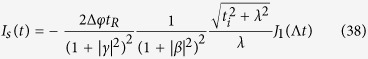


## Additional Information

**How to cite this article**: Caetano, R. A. Spin-Current and Spin-Splitting in Helicoidal Molecules Due to Spin-Orbit Coupling. *Sci. Rep.* **6**, 23452; doi: 10.1038/srep23452 (2016).
